# The Heart of Psoriasis: Cardiovascular Alterations and Common Inflammatory Mechanisms

**DOI:** 10.2174/011573403X412277250911072530

**Published:** 2025-09-25

**Authors:** Elpidio Santillo, Luciano Marini, Lucio Cardinali

**Affiliations:** 1 Rehabilitative Cardiology Unit, Italian National Research Centre on Aging (INRCA-IRCCS), 63900, Fermo, Italy

**Keywords:** Psoriasis, cardiovascular disease, electrocardiography, echocardiography, atherosclerosis, inflammation

## Abstract

**Introduction:**

This mini-review aims to investigate the shared pathophysiological mechanisms linking psoriasis to cardiovascular disease (CVD), with a particular emphasis on electrocardiographic and echocardiographic alterations in individuals affected by psoriasis.

**Methods:**

A comprehensive search of PubMed and Google Scholar was conducted for studies published between January 1980 and June 2025. The search included clinical trials, observational studies, reviews, and meta-analyses.

**Results:**

The pathogenesis of psoriasis shares several key features with CVD, particularly systemic inflammation and endothelial dysfunction. Psoriasis patients frequently exhibit electrocardiographic abnormalities, such as arrhythmias, including atrial fibrillation (AF), and structural cardiac changes, such as left ventricular diastolic dysfunction. These cardiovascular changes are often observed even in the absence of clinically manifest heart disease..

**Discussion:**

Psoriasis significantly contributes to cardiovascular risk, even in patients without manifest CVD. Chronic inflammation, endothelial dysfunction, and metabolic disturbances are key factors contributing to the increased cardiovascular risk observed in these individuals. Furthermore, the presence of psoriatic arthritis may exacerbate these associations, highlighting the multifaceted nature of the disease.

**Conclusion:**

Early detection and management of cardiovascular alterations in patients with psoriasis are essential for mitigating their long-term cardiovascular burden. Targeting shared inflammatory pathways may represent a promising therapeutic strategy to improve both dermatological and cardiovascular health in this patient population.

## 1 INTRODUCTION

Psoriasis is a chronic inflammatory disease that primarily affects the skin, but it can also involve the nails, joints, and, in rarer cases, the gastrointestinal tract and eyes [1]. Over recent years, growing evidence has linked psoriasis to an increased risk of subclinical atherosclerosis, cardiometabolic disorders, and overt cardiovascular events [2-4]. The prevalence of psoriasis in adults varies widely, ranging from 0.51% to 11.43%, depending on geographic and demographic factors [5].

The connection between psoriasis and cardiovascular disease (CVD) was first suggested by the higher prevalence of cardiovascular comorbidities in psoriatic patients. Early studies highlighted that traditional cardiovascular risk factors, such as hypertension, type 2 diabetes, and hypercholesterolemia, are commonly observed in individuals with psoriasis, often as components of metabolic syndrome [6, 7]. This association has since been reinforced by a meta-analysis of 26 observational studies, which included over 17,000 patients with psoriasis and more than 66,000 controls, confirming a significantly higher burden of cardiovascular risk factors in the psoriasis group [8].

Gender-specific analyses have further revealed that women with psoriasis are more likely to exhibit metabolic and cardiovascular risk factors compared to their counterparts without the disease [9]. Longitudinal studies have consistently demonstrated that psoriasis is linked to an increased risk of major cardiovascular events [10, 11], with early disease onset, greater severity, and the presence of psoriatic arthritis being key predictors of this heightened risk [12].

Additionally, psoriasis imposes a considerable economic burden, with healthcare costs higher than those of matched controls, primarily due to the need for more frequent medical visits to manage cardiovascular risk factors, such as diabetes, hypertension, dyslipidemia, and obesity, as well as the increased use of antihypertensive and antidiabetic medications [13]. Notably, patients with severe psoriasis are at an increased risk of out-of-hospital cardiac arrest, a risk not observed in those with milder forms of the disease [14].

The pathogenesis of psoriasis shares several key mechanisms with CVD, driven by a complex interplay of genetic, environmental, immunological, and inflammatory factors, though the precise pathways remain incompletely understood [15]. The term “psoriatic disease” reflects the systemic nature of this inflammation, which extends beyond the skin to affect multiple organs and tissues [16]. Approximately 25% of individuals with psoriasis develop psoriatic arthritis, a heterogeneous inflammatory joint disease that can be mistaken for other forms of arthritis [17, 18].

Importantly, up to 80% of patients with psoriatic arthritis also have concurrent cardiovascular disease [19]. This overlap between psoriasis, psoriatic arthritis, and CVD is largely driven by shared inflammatory mechanisms that contribute to the initiation and progression of atherosclerosis, as well as bioelectrical and structural cardiac remodeling [20]. Cardiac abnormalities in psoriasis, such as mild left ventricular diastolic dysfunction and an increased susceptibility to arrhythmias, often resemble age-related cardiac changes [21].

This review aims to explore the shared pathogenic pathways linking psoriasis to cardiovascular disease, while also summarizing key electrocardiographic and echocardiographic findings in patients with psoriasis, with a particular focus on the cardiovascular implications of psoriatic disease.

## 2 METHODS

### 2.1 Search Strategy

An exhaustive literature search was conducted using PubMed and Google Scholar to identify studies published between January 1980 and June 2025. The search combined MeSH terms and free keywords, including “psoriasis”, “psoriatic arthritis”, “cardiovascular disease”, “atherosclerosis”, “electrocardiography”, and “echocardiography”, using Boolean operators (“AND”, “OR”). Filters were applied to include only studies written in English, involving human subjects, and adult patients (aged 18 years or older). Furthermore, the references of the main articles were reviewed to identify relevant studies not included in the initial search.

### 2.2 Study Selection

Study selection was conducted in two phases: first, screening of titles and abstracts, followed by full-text review. Inclusion criteria comprised clinical studies, cohort studies, case-control studies, observational studies, and narrative or systematic reviews, as well as meta-analyses that addressed cardiovascular pathogenesis or cardiac imaging findings in patients with psoriasis. Exclusion criteria included non-peer-reviewed articles, duplicates, and studies with methodological ambiguities or insufficient data.

### 2.3 Data Extraction and Synthesis

Data were independently extracted by two reviewers using a standardized collection form. The narrative synthesis was organized around the following key themes: shared pathogenic mechanisms and electrocardiographic and echocardiographic abnormalities. Any disagreements in data interpretation were resolved through consensus.

## 3 PATHOGENIC MECHANISMS LINKING PSORIASIS TO CARDIOVASCULAR DISEASE

**Fig. (1):**
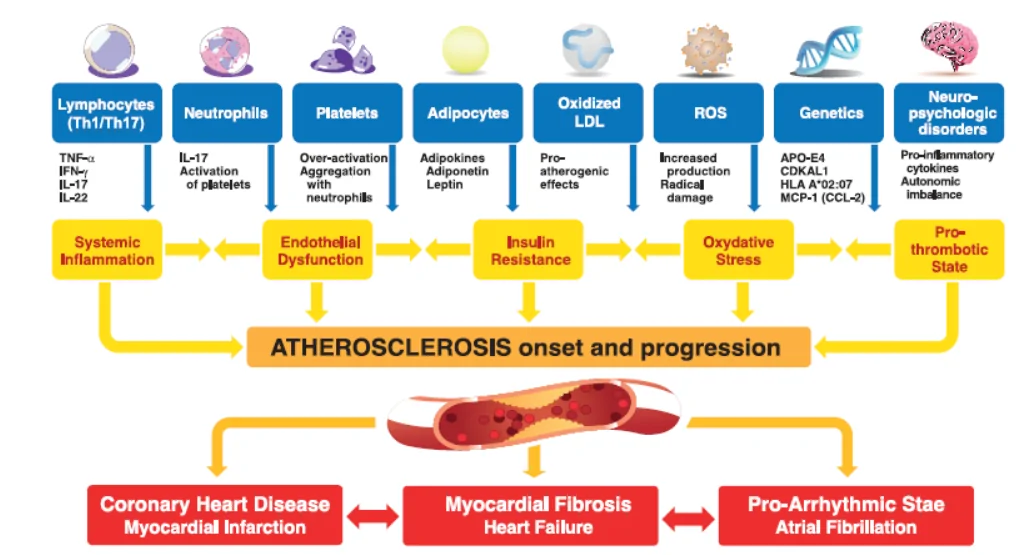
Pathogenic pathways linking psoriasis to cardiovascular disease. This diagram illustrates the complex interconnections between psoriasis and cardiovascular disease. It depicts how cellular and molecular triggers, including activated T lymphocytes (Th1, Th17), neutrophils, platelets, adipocytes, oxidative stress, oxidized LDL, and genetic factors, converge to initiate a cascade of pathophysiological mechanisms. These mechanisms include systemic inflammation, endothelial dysfunction, insulin resistance, oxidative stress, and a prothrombotic state, all of which contribute to the initiation and progression of atherosclerosis.

An increasing understanding of the cellular and molecular pathways involved in both psoriasis and cardiovascular disease (CVD) has revealed a shared pathogenic framework (Fig. **1**) [22]. Chronic inflammation, driven by immune cells, is a central feature of both conditions. In addition, psoriasis and CVD share several vascular and metabolic alterations in their pathogenesis, and recent studies have identified significant genetic correlations between the two.

### 3.1 Key Cellular Actors in Psoriasis and Cardiovascular Disease

Helper T lymphocytes, particularly Th1 and Th17 cells, are critical in the development of psoriasis [23]. These cells are also implicated in atherosclerosis, where interactions between T cells and smooth muscle cells promote plaque formation. In psoriasis, however, the interaction between T cells and keratinocytes is essential for the development of psoriatic plaques. Importantly, Th1 lymphocytes sustain the inflammatory state by producing cytokines such as tumor necrosis factor-alpha (TNF-α), interferon-gamma (IFN-γ), interleukin-2 (IL-2), and adhesion molecules, including soluble intercellular adhesion molecule-1 (sICAM-1). These mediators contribute to the pathogenesis of both psoriasis and CVD [24].

Additionally, Th1 lymphocytes recruit other immune cells, including neutrophils, CD8+ cytotoxic T cells, and macrophages, which further amplify and prolong the inflammatory response. This chronic inflammation leads to excessive fibrin production, disrupting the physiological coagulation balance and predisposing individuals to thrombosis [25].

Th17 lymphocytes are stimulated by the overproduction of IL-23 by keratinocytes and dendritic cells, which in turn promote keratinocyte hyperproliferation through IL-17A and IL-22 signaling [26]. Regulatory T cells (Tregs), which normally suppress inflammation, exhibit an altered skin-trafficking phenotype in psoriasis, impairing their ability to effectively control inflammation in lesional skin [27]. Interestingly, recent studies on the pathogenesis of atherosclerosis have implicated both Th17 lymphocytes and Tregs in the development, instability, and rupture of plaques [28].

Neutrophils also play a pivotal role in both psoriasis and CVD, contributing to vascular inflammation and platelet aggregation through the secretion of interleukin-17 (IL-17) [29, 30]. Platelet hyperactivation represents another critical link between atherosclerosis and psoriasis [31, 32].

### 3.2 Vascular Links

Endothelial dysfunction and insulin resistance-key features of atherogenesis-are commonly observed in patients with psoriasis [33]. Endothelial dysfunction appears to be triggered by the chronic inflammatory state characteristic of psoriasis, mediated by cytokines such as IL-1, IL-6, and TNF-α. Reactive oxygen species (ROS) and tissue hypoxia further contribute to endothelial dysfunction in both psoriasis and CVD [34]. Interestingly, endothelial dysfunction, ROS-induced damage, and T-cell activation are well-established features of cardiac aging [35, 36]. Thus, psoriasis may accelerate cardiovascular aging, supported by findings of shorter telomere length in T cells from psoriasis patients, likely due to increased cell division rates or heightened oxidative stress [37, 38].

### 3.3 Metabolic Links

Insulin resistance plays a significant role in both CVD pathogenesis and the progression of psoriatic disease [39]. In psoriasis, insulin resistance may arise from an imbalance in the adipokine network, which perpetuates chronic inflammation and promotes atherogenesis. This is consistent with the frequent diagnosis of psoriasis in obese individuals, with obesity itself being an independent risk factor for the development of psoriasis [40]. Moreover, elevated homocysteine levels and overexpression of endothelin-1 in psoriasis patients support the hypothesis that endothelial dysfunction and insulin resistance are integral to psoriasis pathogenesis [41, 42].

The altered lipid profile and modifications in the gut microbiome observed in psoriasis patients mirror those seen in CVD, further strengthening the connection between the two conditions [43, 44]. In particular, the gut microbiome is responsible not only for fermentative and absorptive processes related to diet but also, when altered, for triggering abnormal immune responses that release pro-inflammatory cytokines, thereby predisposing individuals to insulin resistance [45]. A new frontier of research in understanding the interplay between psoriasis and cardiovascular health lies in the skin microbiota [46]. For instance, a recent multi-omics study identified antiviral responses and the presence of *Corynebacterium simulans* in the skin microbiome of patients with more severe psoriasis [47]. Similarly, certain bacterial agents, including members of the *Corynebacterium* genus, have been found in atherosclerotic plaques and samples from the human common carotid artery, suggesting they may play a role in the development of cardiovascular diseases [48].

### 3.4 Psychological Links

Psychosocial distress and depression, common in psoriasis patients, can exacerbate systemic inflammation, particularly neuroinflammation, and contribute to increased coronary plaque instability through amygdala hyperactivation [49, 50]. Consequently, individuals with psoriasis are likely to experience negative effects not only emotionally but also on their cardiovascular health. These effects arise from both the heightened stress associated with disease management and the inflammatory processes themselves, mediated by cytokines and neuropeptides [51]. Notably, self-reported depression in psoriasis patients has been linked to increased vascular inflammation and coronary artery disease, as assessed by 18-fluorodeoxyglucose positron emission tomography/computed tomography [52]. These psychological factors, combined with the impact of psoriasis on quality of life, often lead to poor lifestyle choices, further promoting atherogenesis.

### 3.5 Genetic Basis of the Psoriasis-CVD Association

Genetic studies examining the relationship between psoriasis and cardiovascular comorbidities have identified several potential genetic links between the two conditions. For example, the apolipoprotein E-4 (APOE4) gene, which is involved in cholesterol regulation, has been associated with both psoriasis and an increased risk of cardiovascular disease [53]. Similarly, the CDKAL1 gene, a well-established locus in psoriasis, has also been linked to type 2 diabetes [54]. Specific HLA alleles commonly found in psoriasis patients, such as HLA-A02:07 and HLA-C01:02, are further associated with an elevated risk of cardiovascular comorbidities, including hypertension and dyslipidemia [55].

Recent genetic studies have identified shared susceptibility loci between psoriasis and cardiovascular disease, including key polymorphisms such as HLA-Cw6 and IL-23R, both of which modulate immune responses and inflammation [56]. Specifically, the HLA-Cw6 allele appears to exert a protective effect against the development of diabetes mellitus in individuals with psoriatic disease [57].

In contrast, minor variants of IL23R have been linked to an increased risk of type 2 diabetes mellitus [58].

In addition to common inflammatory and immunological pathways, recent studies utilizing microarray data analysis have highlighted 16 hub genes, including MMP9, a member of the matrix metalloproteinase (MMP) family. MMP9 is an endoprotease with collagenase and/or gelatinase activity. These enzymes exert deleterious effects on endothelial integrity and collagen fibers, promoting the destabilization of atherosclerotic plaques and accelerating the process of atherothrombosis [59].

While Genome-Wide Association Studies (GWAS) have shown that the genetic architecture of psoriasis is distinct from, or only modestly related to, that of cardiometabolic diseases [60, 61], the genetic network approach has uncovered shared genes and biological pathways between psoriasis, type 2 diabetes, obesity, and myocardial infarction. For instance, both psoriasis and myocardial infarction are associated with elevated levels of MCP-1/CCL2 (monocyte chemoattractant protein 1/chemokine C-C motif ligand 2), which plays a crucial role in recruiting inflammatory mediators [62]. Additionally, genetic risk factors for cardiovascular disease (CVD) have been linked to an increased risk of psoriasis, but not to other immune-mediated diseases [63].

Furthermore, epigenetic mechanisms, including alterations in DNA methylation patterns, histone modifications, and regulation by non-coding RNAs, have been implicated in both diseases [64, 65]. These mechanisms may mediate gene-environment interactions that contribute to the development of chronic inflammation and atherosclerosis.

Overall, these findings highlight the complex genetic and epigenetic mechanisms linking psoriasis to cardiovascular diseases. While traditional inflammatory pathways remain central to the pathogenesis of both conditions, the complex interplay between genetic variants, epigenetic modifications, and environmental factors may converge on common biological pathways such as inflammation or influence shared cardiovascular risk factors, thereby amplifying the association between these diseases.

## 4 ELECTROCARDIOGRAPHIC ANOMALIES IN PSORIASIS

Multiple studies have shown that patients with psoriasis, particularly those with psoriatic arthritis, often exhibit electrocardiographic abnormalities and a predisposition to arrhythmias (Table **1**). For example, a 2007 study by Markuszeski *et al.* found that patients with psoriasis vulgaris who underwent 24-hour Holter ECG monitoring had significantly higher rates of supraventricular ectopic beats compared to controls, as well as elevated heart rates [66]. In patients with psoriatic arthritis, increased incidences of premature atrial beats and prolonged PR intervals have been reported [67, 68]. A population-based cohort study in Taiwan confirmed an increased arrhythmic risk in patients with psoriasis, independent of traditional cardiovascular risk factors. Interestingly, the risk of arrhythmia was even higher in psoriatic arthritis patients [69].

### 4.1 Psoriasis and Atrial Fibrillation Risk

A nationwide study from Denmark in 2011 identified an association between psoriasis and an increased risk of atrial fibrillation (AF) and ischemic stroke [70]. This finding is further supported by the Losartan Intervention For Endpoint (LIFE) trial, which showed that psoriasis is an independent predictor of new-onset AF in hypertensive patients with left ventricular hypertrophy [71]. However, a more recent prospective study did not confirm a direct link between psoriasis and AF, suggesting that the lack of statistical power in their study may have missed subgroups at higher risk of developing AF [72]. Nonetheless, several electrophysiological anomalies, such as prolonged intra- and inter-atrial conduction time, heterogeneous sinus impulse propagation, prolonged atrial electromechanical coupling intervals, and increased P-wave dispersion, have been noted in psoriasis patients. These abnormalities may predispose them to the development of AF [73-76]. A recent meta-analysis confirmed that psoriasis patients are at significantly higher risk for AF [77].

### 4.2 Inflammation as a Pro-arrhythmic Factor in Psoriasis

Inflammation likely contributes to the arrhythmic burden in patients with psoriasis. Chronic inflammation not only accelerates atherosclerosis but also impairs the function of ion channels in cardiomyocytes [78, 79]. Subclinical inflammation can elevate heart rate and reduce heart rate variability in otherwise healthy individuals [80], suggesting that autonomic dysfunction may also contribute to the increased cardiovascular risk in psoriasis patients [81]. Furthermore, inflammation and subclinical atherosclerosis in psoriasis may alter ventricular repolarization, increasing the risk of ventricular arrhythmias and sudden cardiac death [82, 83].

## 5 ECHOCARDIOGRAPHIC ALTERATIONS IN PSORIASIS

Echocardiographic studies have revealed several abnormalities in patients with psoriasis (Table **2**). Recent advancements in echocardiography, including 3D imaging and speckle tracking, have significantly enhanced our understanding of the structural and functional cardiac alterations in these patients. Even individuals with psoriatic arthritis show notable cardiac changes, likely driven by their chronic inflammatory state. Some of the structural and functional cardiac abnormalities observed in psoriasis may be directly linked to persistent inflammation, which can lead to the development of fibrosis. Autopsy studies have shown that myocardial fibrosis is more pronounced in these patients compared to controls [84]. This increased myocardial fibrosis may partly explain the heightened risk of developing heart failure in psoriasis patients, a risk that is also influenced by genetic factors [85, 86].

### 5.1 Main Echocardiographic Findings in Psoriasis

Early echocardiographic studies consistently reported a significantly higher incidence of left ventricular hypertrophy, diastolic dysfunction, and wall motion abnormalities in psoriasis patients. Additionally, mitral and tricuspid valve prolapse were more common, along with signs of mild pulmonary hypertension and reduced aortic distensibility [87-89]. More recent research by Gorga *et al.* corroborated these findings, showing that young, otherwise healthy individuals with psoriasis exhibited increased left ventricular diastolic dysfunction and mitral regurgitation compared to controls [90]. Furthermore, coronary flow reserve (CFR) is reduced in patients with psoriasis, even in the absence of overt coronary artery disease, suggesting underlying microvascular dysfunction of the heart [91-93]. Importantly, alterations in CFR have significant prognostic value in patients with psoriasis, as they are associated with reduced cardiovascular event-free survival. Evaluating CFR can help identify individuals at higher risk for cardiovascular complications [94]. In line with CFR studies, the coronary artery calcium score, assessed through computed tomography, is significantly elevated in psoriasis patients, suggesting that the development of coronary artery disease may be as prevalent as microvascular dysfunction in these patients [95, 96]. Notably, psoriasis patients also exhibit significantly higher epicardial fat thickness (EFT), a marker increasingly recognized as a novel indicator of cardiometabolic risk [97-99]. EFT, which can be easily measured through transthoracic echocardiography, has been linked to an elevated risk of subclinical atherosclerosis and coronary artery disease [100].

### 5.2 Contributions from Innovative Echocardiographic Techniques

Advances in 3D echocardiography and speckle tracking have allowed for a more detailed assessment of the “psoriatic heart.” For instance, left atrial (LA) volumes were found to be significantly larger in psoriasis patients, with indices of LA mechanical function showing impairment compared to controls. Since LA volume is a well-established predictor of future cardiovascular events, this finding is particularly significant [101]. Additionally, the speckle tracking technique revealed reduced global strain values in psoriasis patients, indicating subtle impairments in left ventricular systolic function that traditional echocardiography failed to detect [102-104]. Similarly, in patients with Metabolic Dysfunction-Associated Steatotic Liver Disease (MASLD), speckle tracking echocardiography demonstrated a significant reduction in left ventricular Global Longitudinal Strain (GLS), an index of myocardial deformation, compared to individuals without MASLD [105]. This suggests that conditions characterized by insulin resistance and chronic inflammation, such as psoriasis and MASLD, can lead to alterations in both the geometry and function of the left ventricle, independent of traditional cardiovascular risk factors. Furthermore, the reduction in left ventricular GLS in psoriasis patients has been associated with a significantly increased risk of adverse cardiovascular events, independent of other cardiovascular risk factors [106].

### 5.3 Echocardiographic Abnormalities in Psoriatic Arthritis

Echocardiographic studies focusing on psoriatic arthritis have identified various cardiac abnormalities, including increased mitral valve prolapse and mild diastolic dysfunction [107, 108]. A more recent narrative review confirmed the higher prevalence of left ventricular diastolic dysfunction in patients with psoriatic disease, particularly those with psoriatic arthritis [109]. These findings support the hypothesis that the chronic inflammatory state associated with psoriasis can lead to morpho-functional myocardial impairments.

## 6 DISCUSSION

This mini-review underscores the growing body of evidence linking psoriasis to an increased risk of atherosclerotic CVD. Even in the absence of clinically overt CVD, psoriasis patients frequently exhibit early cardiac alterations such as mild left ventricular diastolic dysfunction, reduced CFR, and and a heightened burden of arrhythmias, particularly atrial fibrillation. These findings suggest that inflammatory mechanisms play a central role in this association, especially in individuals with severe psoriasis or psoriatic arthritis.

The “psoriatic march,” a conceptual model that describes the progression from chronic systemic inflammation to cardiometabolic complications, has garnered considerable attention in recent years, underscoring the need for integrated cardiovascular evaluation in psoriasis management [110]. This model emphasizes the systemic nature of psoriasis, reinforcing the idea that psoriasis should not be viewed merely as a dermatological disorder, but as a condition with potential multi-organ involvement, including the heart [111]. Major cardiovascular societies now recognize psoriasis as a “risk-enhancing factor” for CVD, advocating for heightened clinical vigilance and early identification of cardiovascular abnormalities, even in asymptomatic patients [112].

As a result, clinicians need to adopt a more comprehensive approach to managing psoriasis. Lifestyle modifications such as regular physical activity, adherence to a Mediterranean-style diet, and smoking cessation should be strongly encouraged, as these interventions benefit both psoriasis and cardiovascular health [113, 114].

Emerging evidence suggests that pharmacological treatments used in the management of psoriasis may also have a positive influence on cardiovascular risk profiles. Methotrexate, for instance, has been associated with a reduction in cardiovascular events, although it may elevate homocysteine levels [115-117]. Among biologic therapies, TNF-α inhibitors, including etanercept, infliximab, and adalimumab, commonly used for the treatment of moderate to severe psoriasis, have shown promise in mitigating systemic endothelial dysfunction, reducing vascular inflammation, and slowing the progression of atherosclerosis [118, 119]. While these agents appear effective in reducing cardiovascular events in patients with psoriasis, the benefits may vary among individuals [120-122]. Further large-scale longitudinal studies are necessary to gain a deeper understanding of long-term outcomes.

Additionally, novel biologics targeting the IL-17 and IL-23 pathways, which are critical mediators in psoriatic inflammation, show potential in improving cardiovascular outcomes [123]. The IL-17/23 axis plays a key role in modulating the chronic low-grade inflammatory state, which may help explain the association between psoriasis and metabolic comorbidities. Accordingly, expert consensus supports the use of anti-IL-17 and anti-IL-23 therapies as first-line treatments for psoriasis patients at high risk for cardiovascular diseases (CVDs) or metabolic syndrome [124].

Recent imaging studies further suggest that biologics may also slow the progression of coronary artery plaques [125]. These findings enhance the biological plausibility of a link between psoriasis and cardiovascular disease, with the cardioprotective effects likely driven by the anti-inflammatory properties of these treatments [126]. Certain cardiovascular drugs, such as statins, may also provide protective effects in psoriasis, potentially due to their anti-inflammatory mechanisms [127].

Despite these valuable insights, the present review has several limitations. Unlike systematic reviews, it does not follow a predefined, registered protocol for literature search and study selection, which may introduce selection bias. The synthesis is based on qualitative analysis influenced by the authors’ expertise and interpretation. Additionally, significant heterogeneity exists among the studies referenced, particularly in terms of population characteristics, diagnostic criteria, and outcome measures, limiting the generalizability of our conclusions. The lack of a quantitative synthesis, such as a meta-analysis, prevents the estimation of pooled effect sizes and the drawing of statistically robust conclusions. Furthermore, publication bias and the absence of formal quality appraisal for the included studies may limit the strength of the evidence presented.

Nevertheless, this review provides a concise yet comprehensive overview of current knowledge, highlighting clinically relevant cardiovascular findings that are often underrecognized in psoriasis care. It also provides practical recommendations for clinicians, advocating for the integration of cardiovascular screening and lifestyle counseling into routine care for patients with psoriasis.

## CONCLUSION

Psoriasis is increasingly recognized as a systemic inflammatory disease with significant cardiovascular implications. Even in the absence of overt heart disease, patients with psoriasis often exhibit subclinical cardiac abnormalities and are at heightened cardiovascular risk. Clinicians should adopt a multidisciplinary approach, incorporating routine cardiovascular screening, such as ECG and echocardiography, when appropriate, into the management of moderate to severe psoriasis.

Given the shared inflammatory mechanisms between psoriasis and cardiovascular disease, lifestyle modifications, including regular physical activity, a heart-healthy diet, and smoking cessation, should be actively encouraged.

Special attention should be given to high-risk individuals, including those with long-standing disease, psoriatic arthritis, or extensive skin involvement. In these patients, early referral to cardiology and proactive management of risk factors may help mitigate long-term cardiovascular complications.

Future research should focus on understanding the effects of psoriasis-specific therapies on cardiovascular outcomes. Longitudinal studies are necessary to evaluate the progression of subclinical cardiac changes and their potential reversibility with anti-inflammatory therapies. Additionally, the identification of novel biomarkers and imaging tools for cardiovascular risk stratification in patients with psoriasis could help refine personalized care strategies.

By recognizing psoriasis as a multisystem disease with far-reaching implications, clinicians can improve long-term outcomes and reduce cardiovascular morbidity and mortality in this population.

## Figures and Tables

**Table 1:** Electrocardiographic anomalies in psoriasis patients.

**Electrocardiographic Anomaly**	**Description**	**References**
Supraventricular ectopic beats	Higher frequency of ectopic beats in psoriasis patients compared to controls	[66]
Heart rate	Increased heart rate in psoriasis patients	[66]
Premature atrial beats	Increased incidence in patients with psoriatic arthritis	[67, 68]
Prolonged PR interval	Seen more frequently in psoriatic arthritis patients	[67, 68]
Atrial fibrillation risk	Higher risk of atrial fibrillation in psoriasis patients, independent of traditional risk factors	[70, 71]
Intra and inter-atrial conduction time	Prolonged intra- and inter-atrial conduction time in psoriasis patients	[73-76]
P-wave dispersion	Increased P-wave dispersion, which can predispose to atrial fibrillation	[73-76]
Arrhythmia risk	Elevated risk of arrhythmia confirmed by meta-analysis	[77]
Inflammation as a pro-arrhythmic factor	Inflammation could impair ion channel function and increase heart rate variability	[78-80]
Autonomic dysfunction	Impaired autonomic regulation, contributing to arrhythmic risk	[81]

**Table 2:** Echocardiographic abnormalities in psoriasis patients.

**Echocardiographic Abnormality**	**Description**	**References**
Left ventricular hypertrophy	Increased frequency of left ventricular hypertrophy in psoriasis patients	[87]
Diastolic dysfunction	Higher incidence of left ventricular diastolic dysfunction	[87, 90]
Mitral and tricuspid valve prolapse	Increased occurrence of mitral and tricuspid valve prolapse in psoriasis patients	[87]
Pulmonary hypertension	Mild pulmonary hypertension is seen more frequently in psoriasis patients	[88, 89]
Aortic distensibility	Decreased aortic distensibility was observed in psoriasis patients	[88, 89]
Coronary flow reserve (CFR)	Reduced CFR in psoriasis patients, even without coronary disease	[91, 92]
Epicardial fat thickness (EFT)	Increased EFT, associated with higher cardiovascular risk and subclinical atherosclerosis	[97-99]
Left atrial volume	Higher left atrial volume in psoriasis patients, predictive of future cardiovascular events	[101]
Left ventricular strain	Reduced left ventricular strain in psoriasis patients, suggesting impaired systolic function	[102-104]
Mitral regurgitation	Increased mitral regurgitation in young and healthy individuals with psoriasis	[90]

## LIST OF ABBREVIATIONS

AF: Atrial Fibrillation
APOE4: Apolipoprotein E-4
CDKAL1: Cyclin Dependent Kinase Like 1
CVD: Cardiovascular Disease
ECG: Electrocardiogram
EFT: Epicardial Fat Thickness
GLS: Global Longitudinal Strain
HLA: Human Leucocyte Antigen
IFN-γ: Interferon Gamma
IL: Interleukin
LDL: Low-Density Lipoprotein
LVEF: Left Ventricular Ejection Fraction
MASLD: Metabolic Dysfunction-Associated Steatotic Liver Disease
MCP-1/CCL2: Monocyte Chemoattractant Protein-1 / Chemokine C-C Motif Ligand 2
PASI: Psoriasis Area Severity Index
ROS: Reactive Oxygen Species
sICAM-1: Soluble Intercellular Adhesion Molecule-1
Th1: T Helper Type 1
Th17: T Helper Type 17
TNF-α: Tumor Necrosis Factor Alpha
Tregs: regulatory T cells
